# Fertility and Obstetric Outcomes of Assisted Reproductive Technology (ART) in Women With Adenomyosis Following Gonadotropin-Releasing Hormone Agonist Therapy: A Single-Center Experience

**DOI:** 10.7759/cureus.44691

**Published:** 2023-09-04

**Authors:** Asha R Rao, Uthra Priyadarshini Duraikannan, Damodar R Rao, Padmashri Giridhar, Sivakumar N Chinnasamy

**Affiliations:** 1 Department of Assisted Reproductive Technology, Rao Hospital, Coimbatore, IND

**Keywords:** gonadotropin-releasing hormone agonist, obstetric outcomes, live birth rate, in vitro fertilization, ovarian stimulation, adenomyosis

## Abstract

Introduction: Adenomyosis is an ambiguous disorder causing a wide variety of implications from dysmenorrhea, heavy menstrual bleeding, and infertility to pregnancy complications. Adenomyosis is associated with altered endocrine and inflammatory milieu, resulting in impaired implantation and reduced fertility potential. It is also associated with increased incidence of obstetric complications such as miscarriage, antepartum hemorrhage, placental mal-position, hypertensive disorders, small for gestational age-intrauterine growth restriction (SGA-IUGR), cesarean section, preterm labor, preterm premature rupture of membranes (PPROM), and neonatal intensive care unit (NICU) admissions.

Objective: The aim of our study was to investigate the fertility and obstetric outcomes in women with adenomyosis treated with GnRH agonists compared to controls with normal uteri undergoing in-vitro fertilization (IVF) at our center, thereby establishing the role of gonadotropin-releasing hormone (GnRH) agonists in managing sub-fertile women with adenomyosis.

Materials and methods: We carried out a retrospective cohort study at our hospital to analyze the effects of adenomyosis on IVF and pregnancy outcomes. This study (n=83) involves women with adenomyosis between the ages of 21 and 37 years who were followed up at our center between 2013 and 2022. The controls (n=83) were selected from women who underwent IVF-intracytoplasmic sperm injection (IVF-ICSI) for tubal or mild male factor infertility with normal appearing uterus within the same time frame. Women with adenomyosis were given GnRH agonist as long/ultralong agonist protocol before controlled ovarian stimulation or as down-regulated frozen embryo transfer (FET). The length of suppression was between one and six months based on the size of the uterus and response to treatment. Fertility and obstetric outcomes were analyzed.

Results: The implantation rates were found to be equivocal: 54.2% and 53% in the adenomyosis and control groups, respectively (p=0.208). The cumulative live birth rate was 50.6% and 48.2% in the study and control groups, respectively (p=0.341). The biochemical pregnancy rate and the first- and second-trimester miscarriage rates were not significantly different between the group with adenomyosis and the group with normal uterus. The incidence of preterm deliveries and antepartum hemorrhage was found to be significantly increased in the study group.

Conclusion: Medical management in women with adenomyosis optimizes the live birth rates giving results at par with the control population.

## Introduction

Adenomyosis is an ambiguous disorder causing a wide variety of implications from dysmenorrhea, heavy menstrual bleeding, and infertility to pregnancy complications [1]. Adenomyosis is characterized by the displacement of endometrial epithelial and stromal cells into the myometrium, causing inflammation, surrounding myo-hyperplasia, and fibrosis [2]. The incidence of adenomyosis seems to be on the rise in the infertile population due to the postponement of pregnancy by women and better imaging modalities for diagnosis [3]. The prevalence of adenomyosis ranges from 7% and 27% [4].

Adenomyosis is associated with altered endocrine and inflammatory milieu, resulting in impaired implantation and reduced fertility potential [5,6]. It is also associated with increased incidence of obstetric complications such as miscarriage, antepartum hemorrhage, placental mal-position, hypertensive disorders, small for gestational age-intrauterine growth restriction (SGA-IUGR), cesarean section, preterm labor, preterm premature rupture of membranes (PPROM), and neonatal intensive care unit (NICU) admissions [7].

The aim of our study was to investigate the fertility outcomes and obstetric outcomes in women with adenomyosis treated with GnRH agonists compared to controls with normal uteri undergoing in-vitro fertilization (IVF) at our center, thereby establishing the role of gonadotropin-releasing hormone (GnRH) agonists in managing sub-fertile women with adenomyosis.

## Materials and methods

We carried out a retrospective cohort study at our hospital to analyze the effects of adenomyosis on IVF and pregnancy outcomes. This study involves women with adenomyosis between the ages of 21 and 37 years who were followed up at our center between 2013 and 2022. They are considered the study group.

Diagnostic criteria for adenomyosis included at least three of the following - enlarged uterus, heterogeneous myometrium, asymmetry of anterior and posterior myometrium, myometrial cysts, linear striations, ill-defined endometrial-myometrial interface, hyperechogenic islands, and echogenic sub endometrial lines or buds [8,9].

The controls were selected from women who underwent IVF-ICSI for tubal or mild male factor infertility with normal appearing uterus within the same time frame. The control group must have had an ultrasound at our center showing normal myometrial echoes, excluding adenomyosis.

Eighty-three women with ultrasound showing features of adenomyosis were identified between 2013 and 2022 at our hospital, and 83 women were included in the control group. All the ultrasounds were done by one experienced sonographer with 25 years of experience to avoid observer bias. The baseline characteristics analyzed were age, body mass index (BMI), presence of dysmenorrhea and dyspareunia, uterine length, and uterine volume.

The stimulation protocol for controlled ovarian stimulation was a luteal-phase agonist protocol or antagonist or ultralong agonist protocol based on the ovarian reserve and other factors for infertility. Women with adenomyosis were given GnRH agonist as a long/ultralong agonist protocol before controlled ovarian stimulation or as dow-regulated frozen embryo transfer (FET) before embryo transfer. The length of suppression was between one and six months based on the size of the uterus and response to treatment.

The dose of gonadotropins was decided based on age, BMI, ovarian reserve, and associated fertility factors. Fertility outcomes analyzed were implantation rate for the first embryo transfer, cumulative live birth rate (up to two embryo transfers), biochemical pregnancy rate, and first- and second-trimester miscarriage rates.

The obstetric outcomes of the singleton pregnancies analyzed were complications such as pre-term delivery, PPROM, mal-presentation, antepartum hemorrhage (antepartum hemorrhage is defined as bleeding from or into the genital tract occurring between 24+0 weeks' gestation until birth), placenta previa, SGA-IUGR, pregnancy-induced hypertension, and NICU admission.

Ethical committee opinion was obtained from the Institutional Human Ethics Committee, PSG Institute of Medical Sciences & Research (Project No. 23/074).

Statistical tools

The following are the statistical tools used to analyze and interpret the data to arrive at a conclusion. Data are entered into Microsoft Excel and analyzed using Statistical Product and Service Solutions (SPSS) software (version 26.0; IBM SPSS Statistics for Windows, Armonk, NY). Standard statistical procedures of descriptive and inferential statistics were used for the analysis of the data.

Descriptive analyses, such as mean, standard deviation, and percentage, were used to express quantitative variables of clinical parameters considered in the research interpretation. After the data were tested for normality of distribution, the statistical tests were allotted. The Independent sample t-test and chi-square test were used to compare the groups. For interpretation of the results, a p-value of ≤0.05 was considered statistically significant. Qualitative data were described as frequencies (number of cases) and percentages.

## Results

Baseline characteristics (Table 1), such as age and BMI, were comparable between the groups. The antral follicle count was in a better trend in the control group, but it did not reach statistical difference (p=0.074). Anti-mullerian hormone (AMH) was better in the control group. The incidence of dysmenorrhea (p=0.000) and dyspareunia (p=0.001) in the study group was significantly higher. The mean uterine length (p=0.000) and the mean uterine volume (p=0.000) were higher in the adenomyotic uterus before treatment. The ovarian stimulation characteristics such as dose of gonadotropins and estradiol levels were non-significant between the groups. The number of days of stimulation was found to be longer in the adenomyosis group (p=0.002). The number of follicles greater than 15 mm on the day of trigger was more in the study group, but the number of oocytes retrieved remained comparable. The ovarian response parameters and embryological parameters such as the number of oocytes retrieved, follicular output rate (FORT), follicle-to-oocyte index (FOI), fertilization rate, metaphase 2 oocytes, pro-nuclei stage, and number of embryos available for transfer were similar between the groups.

**Table 1:** Basic and stimulation characteristics BMI - Body Mass Index, AMH - Anti-Mullerian Hormone, AFC - Antral Follicle Count, E2 - Sr. Estradiol, M2 - Metaphase 2 oocytes, FORT - Follicular Output Rate, FOI - Follicle-to-Oocyte Index, VI - Vascularization Index, FI - Flow Index, VFI - Vascularization Flow Index

Variables	Study Group (n=83)	Control Group (n=83)	p-value
Age (years)	32.86±4.76	30.99±4.18	0.143
BMI (kg/m^2^)	26.17±3.94	25.51±3.87	0.798
Dysmenorrhoea	50 (60.2%)	13 (16.2%)	0.000
Dyspareunia	10 (12.0%)	0 (0.0)	0.001
Average length of the uterus (cm)	7.67±1.07	6.57±0.67	0.000
Average uterine volume (cc)	85.74±38.51	47.59±15.02	0.000
AMH (ng/ml)	2.08±1.81	3.95±3.30	0.000
AFC	11.49±9.54	20.63±12.34	0.074
Total No. of days of stimulation (days)	9.23±2.05	8.01±1.36	0.002
No. of follicles ≥15 mm on the day of the trigger	5.65±2.64	7.19±6.28	0.052
E2 on the day of trigger (pg/ml)	2027.19±1136.85	2177.59±1318.48	0.342
No. of oocytes retrieved	7.58±4.11	8.29±5.17	0.152
No. of M2 oocytes	5.79±2.67	5.88±3.24	0.127
No. of oocytes fertilized	4.40±2.15	4.44±2.11	0.353
Fertilization rate (%)	75.84±16.76	60.56±17.89	0.925
FORT (%)	44.98±19.37	37.70±19.72	0.819
FOI(%)	0.88±2.21	0.48±0.32	0.084
Endometrial volume (cc)	3.51±1.71	3.54±3.80	0. 416
VI	4.16±5.55	5.67±11.89	0.223
FI	25.18±4.47	21.95±6.05	0.779
VFI	1.22±1.87	1.49±3.55	0.468

The implantation rate (Table 2) was calculated for the first cycle of embryo transfer, and the rates were found to be comparable to 54.2% and 53% in the adenomyosis and control groups, respectively (p=0.208). The cumulative live birth rate was 50.6% and 48.2% in the study and control groups, respectively (p=0.341). The biochemical pregnancy rate and first- and second-trimester miscarriage rates were not significantly different between the group with adenomyosis and the group with a normal uterus.

**Table 2:** Fertility outcomes LBR - Live Birth Rate

	Study Group (n=83)	Control Group (n=83)	p-value
Implantation rate (1^st^ embryo transfer)	45	44	0.208
	54.2%	53.0%	
Cumulative LBR (1^st^ + 2^nd^ embryo transfer)	42	40	0.341
	50.6%	48.2%	
First-trimester miscarriage	6	7	0.773
	7.2%	8.4%	
Biochemical pregnancy	0	2	-
	0.0%	2.4%	
Second-trimester loss	4	3	0.973
	4.8%	3.6%	

Table 3 shows the various obstetric outcomes of singleton pregnancies in the adenomyosis and control groups. The incidence of preterm deliveries and antepartum hemorrhage was found to be significantly increased in the study group. In our study, antepartum hemorrhage was due to abruptio placenta in one case (grade 0), two due to placenta previa, and another two unexplained etiology. PPROM, mal-presentation, and placental disorders such as pregnancy-induced hypertension, SGA-IUGR, and placenta previa were not different between the adenomyosis and control groups.

**Table 3:** Obstetric outcomes for singleton pregnancies in the adenomyosis and control groups PROM - Preterm Premature Rupture of Membrane, APH - Antepartum Hemorrhage, SGA - Small for Gestational Age, IUGR - Intra-Uterine Growth Restriction, NICU - Neonatal Intensive Care Unit

	Study group N=31	Control group N=34	p-value
Preterm delivery	20 (64.5%)	12 (35.3%)	0.019
Pregnancy-induced hypertension	6 (20.0%)	5 (15.2%)	0.613
Placenta previa	2 (6.7%)	0 (0.0%)	0.138
PPROM	4 (13.3%)	4 (12.1%)	0.885
APH	5 (16.7%)	0 (0.0%)	0.015
SGA/ IUGR	5 (16.7%)	9 (27.3%)	0.312
Mal presentation	1 (3.2%)	1 (3.1%)	0.982
NICU admission	12 (38.7%)	6 (18.8%)	0.080

## Discussion

Adenomyosis is associated with adverse fertility and pregnancy outcomes [10]. In our study, we show that medical management before assisted reproductive technology (ART) in women with adenomyosis yields similar fertility outcomes to women with a normal-appearing uterus.

Sharma et al., in a retrospective study, showed that the number of metaphase 2 oocytes and the number of embryos available for transfer were not affected in adenomyosis. They also reported that, when comparing women undergoing ART for tubal factor, endometriosis, and adenomyosis, women with adenomyosis had a lower clinical pregnancy rate, increased miscarriage rate, and lower live birth rate [11].

Deuholm, in her recent meta-analysis, states decreased pregnancy rate (RR 0.73 (95% CI 0.64-0.82)) and live birth rate (RR 0.69 (95% CI 0.56-0.85)) in women with adenomyosis undergoing IVF. Additionally, the miscarriage rate was more prevalent (RR 2.12 (95% CI 1.20- 3.75)) in women with adenomyosis [12].

Horton et al., in their meta-analysis, concluded that adenomyosis is associated with reduced implantation rate (OR 0.56, 95% CI 0.39-0.8, p=0.001; n=3), clinical pregnancy rate (OR 0.57, 95% CI 0.43-0.76, p<0.001; n=7), live birth rate (OR 0.45, 95% CI 0.24-0.86, p=0.02; n=5), and increased risk of miscarriage (OR 3.49, 95% CI 1.41-8.65, p=0.007; n=6). The meta-analysis on pregnancy outcomes showed higher risk of pre-term delivery (OR 2.74, 95% CI 1.89-3.97, p<0.001; n=5), SGA (OR 3.90, 95% CI 2.10-7.25, p<0.001; n=2), cesarean section (OR 2.62, 95% CI 1.00-6.89, p=0.05; n=3), and pre-eclampsia (OR 7.87, 95% CI 1.26-49.20, p=0.03; n=2) [13].

French et al., in their study, have reported that adenomyosis is associated with adverse reproductive outcomes, and treatment with GnRH analogs before FET may increase pregnancy rates. Medical management gives the additional benefit of avoiding the dreaded complication associated with surgery, namely uterine rupture [14].

Adenomyosis is perpetuated by the vicious cycle involving local hyper-estrogenism, increased peristalsis, micro-trauma, and the activation of the tissue repair mechanism, which once again increases estrogen [15,16]. This cycle is interrupted by GnRH agonist, leading to a hypoestrogenic state, which leads to regression of adenomyotic lesions and reduction in uterine volume, enhances endometrial receptivity, and provides immunological suppression, thus improving the endometrial function and fertility outcomes [17].

Various theories have been put forward to explain the better fertility outcomes following GnRH agonist down-regulation. An improved implantation window was suggested by Yoldemir [4].

Khan et al., in their study, have demonstrated that heat shock protein (HSP) 70, which is produced in response to stress, is involved in inflammation, and the growth of endometriosis was also found to be increased in adenomyotic lesions, and they also demonstrated that HSPs reduced following GnRH agonist treatment [18].

The positive outcome of using GnRH agonist pretreatment before ART in women with adenomyosis was demonstrated by two retrospective controlled studies, which compared GnRH agonist pretreatment and no treatment before fresh embryo transfer [19] and FET [20].

Hou et al., in their observational cohort study, showed that women with adenomyosis had better clinical pregnancy rates ((OR 1.925, 95% CI 1.137-3.250, p=0.015), implantation rates (OR 1.694, 95% CI 1.006-2.854, p=0.047), and live birth rates (OR 1.704, 95% CI 1.012-2.859, p=0.044) when they receive ultralong GnRH agonist protocol when compared to the luteal GnRH agonist protocol [21].

Women with larger uterine length and volume received longer periods of GnRH agonist suppression, thus giving almost similar outcomes in women with huge adenomyosis. A longer period of suppression was associated with more reduction in uterine volume and a reduction in the number of ultrasound features of adenomyosis. In a study by Li et al., it was found that uterine volume >98.81 cc was associated with a similar implantation rate but increased miscarriage rate, leading to a decreased live birth rate when compared to women with adenomyosis having a uterine volume of <98.81 cc [22].

Chen et al., in their recent retrospective study, have shown that ultralong GnRH agonist protocol is not associated with better live birth rates when compared to long agonist protocol for stimulation as the supra-physiological estrogen levels during stimulation stimulate the adenomyotic lesions, thus nullifying the results of long agonist pre-treatment [23].

Wu et al., in their study of 537 women with adenomyosis that GnRH agonist down-regulation followed by FET, was associated with significantly better implantation and live birth rates and lower miscarriage rates when compared to fresh embryo transfer following an ultralong GnRH agonist protocol for ovarian stimulation. Additionally, the dose of stimulation and the number of days of stimulation were lower in the deferred transfer group [24].

In our study, our findings were that the implantation rates, miscarriage rates, and cumulative pregnancy rates were comparable between both groups. This was possible even in uteri with adenomyosis as the patients underwent suppression before embryo transfer by the GnRH agonist. The low miscarriage rates were also possible because of the down-regulation. The stimulation characteristics were similar between both groups in accordance with the fact that ovarian response is not affected by uterine adenomyosis.

Obstetric outcomes such as pre-term delivery and antepartum hemorrhage were increased in spite of adjusting for singleton pregnancies in the adenomyosis group in our study. Hashimoto et al. demonstrated that, even after adjusting for confounding factors such as age, primiparity, and ART use, obstetric complications such as preterm delivery, SGA infants, cesarean section, late spontaneous abortion, abnormal placental positioning, and hypertensive disorders of pregnancy were increased in pregnant women with adenomyosis (n=49) when compared to non-adenomyotic women [25].

Limitations of our study are that it is a retrospective study, and it extends over a long study period. However, the cases were handled by a single tertiary care unit where the uniformity of management was maintained as much as possible. Additionally, the history of estrogen/progesterone pill intake and co-morbidities were not assessed in our study.

## Conclusions

In conclusion, medical management in women with adenomyosis undergoing ART provides promising live birth rates similar to women without adenomyosis. The GnRH agonist before frozen embryo transfer is the wiser choice for good outcomes in adenomyosis and women with poor ovarian reserve. Women with a larger uterus require longer periods of suppression. Uterine volume can be used as a guide to plan the duration of medical management.

Obstetric complications should be borne in mind while managing these patients to optimize the outcomes. Women undergoing ART should be screened for adenomyosis, particularly in women with symptoms such as dysmenorrhea, menorrhagia, recurrent implantation failure, and recurrent pregnancy loss. Early identification of adenomyosis helps in the proper management of these women with GnRH agonists, yielding good results. Combined treatment with GnRH agonist and aromatase inhibitor may be tried in huge adenomyosis. More studies are required to substantiate the same.
